# “Who am I?”: Weakened sense of the self in patients with behavioral variant frontotemporal dementia

**DOI:** 10.1097/MD.0000000000033461

**Published:** 2022-04-07

**Authors:** Mohamad El Haj, Dimitrios Kapogiannis, Claire Boutoleau-Bretonnière

**Affiliations:** a Nantes Université, Univ Angers, Laboratoire de Psychologie des Pays de la Loire, LPPL, Nantes, France; b CHU Nantes, Clinical Gerontology Department, Nantes, France; c Institut Universitaire de France, Paris, France; d Laboratory of Clinical Investigation, National Institute on Aging, Baltimore, MD; e CHU Nantes, Inserm CIC04, Nantes, France.

**Keywords:** behavioral variant frontotemporal dementia, identity, self, sense of the self

## Abstract

While research has shown a distrusted sense of the self in patients with behavioral variant frontotemporal dementia (bvFTD), little is known about how patients describe their self-image. We used the “Who am I?” task to invite patients with bvFTD and control participants to produce statements beginning with “I am….” We distinguished between statements related to physical, social, and psychological self. Analyses showed fewer statements related to physical, social, and psychological self in the patients with bvFTD than in control participants. Another result was the proportionally similar production of statements describing physical, social, and psychological self in both patients with bvFTD and control participants. Finally, the total production of “Who am I?” statements was positively correlated with verbal fluency in both patients with bvTFD and control participants. Our findings demonstrate a diminished ability of patients with bvFTD to process self-images. Our study also paves the way toward the use of the “Who am I” task as a simple and ecologically valid tool allowing for the quantitative and qualitative assessment of the sense of self in patients with bvFTD.

## 1. Introduction

The concept of the self has proved particularly difficult to define because the self-embeds a set of multidimensional and dynamic processes that support, among other dimensions, consciousness, identity, and agency.^[1,2]^ However, the self can be widely defined as the processes that provide us with feelings of intimacy and coherence that define our uniqueness.^[3,4]^ A distinction between various processes that support the self has been proposed by Charlesworth, Allen, Havelka and Moulin^[5]^ who distinguished between physical, social, and psychological self. This distinction, inspired from the concepts of William James,^[6]^ considers the physical self as the knowledge about attributes related to our own appearance and physical condition (e.g., I am small, I am sick), social self as the knowledge about our own social category (e.g., I am retired), and psychological self as the knowledge about our own personality traits (e.g., I am kind). The physical, social, and psychological self can be evaluated with the “Who am I” task.^[7]^ On this easy-to-administer and easy-to-score task, participants are typically invited to provide self-images by responding open-endedly to the question “Who am I?.” This task allows reliable evaluation of the processes that support factual and evaluative knowledge of one’s self (e.g., “I am healthy,” “I am sick”…).^[8]^ Using this task, the present paper evaluates how patients with behavioral variant frontotemporal dementia (bvFTD) define and describe their physical, social, and psychological self.

We investigated how patients with bvFTD define themselves because bvFTD is characterized not only by changes in behavior, social cognition, and personality, but also by changes in the sense of self.^[9,10]^ The sense of self in patients with bvFTD has been mainly assessed regarding anosognosia, although the sense of self differs from anosognosia as the sense of self refers to the subjective view of who we are while anosognosia refers to the patients difficulties to appraise their owns cognitive or neurologic impairments.^[11–14]^ For instance, Eslinger, Dennis, Moore, Antani, Hauck, and Grossman^[15]^ assessed anosognosia regarding behavioral changes in a mixed frontotemporal lobar degeneration (FTLD) group (i.e., the umbrella term, combining bvFTD and language-predominant frontotemporal neurodegenerative diseases), and observed a tendency of patients to underestimate their symptoms (e.g., apathy), in comparison to their caregivers complaints regarding these symptoms. In another study using a mixed FTLD group, O’Keeffe, Murray, Coen, Dockree, Bellgrove, Garavan, Lynch and Robertson^[16]^ assessed patients awareness regarding their medical situation, their online monitoring of their own errors, as well as their ability to accurately predict their own performance on cognitive tasks. Results demonstrated anosognosia in all assessed domains. Anosognosia in bvFTD was also reported by Ruby, Schmidt, Hogge, D’Argembeau, Collette, and Salmon^[17]^ who invited patients with bvFTD to describe how they would probably react in situations likely to trigger emotional reactions (e.g., ‘“you are late for an appointment, how do you react?”). Patients were also invited to indicate how well adjectives describe their personality. Results demonstrated impaired ability of patients in both predicting their own behavior and describing their personality. Anosognosia in bvFTD can be attributed to several factors, such as deficits in online monitoring and response to environmental feedback^[18]^ and impaired affective processing in general.^[19]^ Taken together, the sense of self in bvFTD has been extensively assessed regarding self-awareness, and research in this domain has documented the impaired ability of patients in recognizing, describing and appraising their own behavioral and cognitive deficits.

The disruption in the sense of the self in bvFTD can be illustrated by shedding light on research on self-conscious emotional processes. In a study by Sturm, Rosen, Allison, Miller, and Levenson,^[20]^ patients with FTLD and contols were exposed suddenly to a loud noise. The authors assessed the reflexive responses (i.e., the muscle contractions in the face and upper torso) as well as the self-conscious responses (i.e., how the participants process/comment on their own reactions). While no differences were observed between patients with FTLD and control participants regarding reflexive response, self-conscious responses were markedly diminished in the patients. In a similar study, Sturm, Ascher, Miller, and Levenson^[21]^ assessed self-conscious emotional responses by inviting patients with FTLD, in a karaoke singing task, to sing alone. Participants were filmed during the task and, afterwards, they were invited to hear and see themselves singing. Participants physiological responses (e.g., skin conductance), were recorded when watching themselves singing. Results demonstrated diminished self-conscious emotional responses (e.g., embarrassment), as well as diminished physiological responses in the patients while watching themselves singing.

To summarize, while research has assessed the self in bvFTD, little is known about how the patients process their physical, social, and psychological self. We thus carried out this study to demonstrate how the “Who am I” task can be used as a simple and ecological tool to quantitatively and qualitatively explore the 3 categories of self in bvFTD. We also carried out this study to understand which self-component (i.e., physical, social, and psychological self) is preserved, or impaired, in bvFTD. In our view, such investigation may reveal which self-component is more of less salient for patients. We thus invited patients with bvFTD and control participants perform the “Who am I?” task. Besides comparing the quantity of statements between patients with bvFTD and control participants, we were interested by the qualitative distinction between statements describing physical self, social self, or psychological self in each population. We hypothesized that, compared to control participants, patients with bvFTD would produce less statements describing physical, social, and psychological self.

## 2. Method

### 2.1. Participants

The sample consisted of 13 patients (8 men and 5 women, *M* age = 65.89 years, *SD* = 6.45, *M* years of formal education = 10.31, *SD* = 4.72) meeting criteria for bvFTD as defined by Rascovsky, Hodges, Knopman, Mendez, Kramer, Neuhaus, van Swieten, Seelaar, Dopper, Onyike, Hillis, Josephs, Boeve, Kertesz, Seeley, Rankin, Johnson, Gorno-Tempini, Rosen, Prioleau-Latham, Lee, Kipps, Lillo, Piguet, Rohrer, Rossor, Warren, Fox, Galasko, Salmon, Black, Mesulam, Weintraub, Dickerson, Diehl-Schmid, Pasquier, Deramecourt, Lebert, Pijnenburg, Chow, Manes, Grafman, Cappa, Freedman, Grossman, and Miller.^[9]^ The inclusions criteria for patients with bvFTD were thus the presence of at least 3 of 6 core features (disinhibition, apathy, loss of sympathy and/or empathy, perseverative and/or compulsive behaviors, hyperorality and dietary changes, and executive dysfunction), functional impairment by Activity of Daily Living, and imaging results suggesting frontal and/or anterior temporal atrophy. Exclusions criteria were other forms of dementias, as well as other forms of frontotemporal dementias such as primary progressive aphasia.

The sample also included 15 control participants (10 men and 5 women, *M* age = 66.27 years, *SD* = 6.65, *M* years of formal education = 10.56, *SD* = 4.82). Patients with bvFTD (*M* years since diagnosis = 2.11, *SD* = 1.05) were mainly referred by general practitioners and neurologists in the Nantes Memory Center. We recruited the control participants from local community. They were free of any neurological/psychiatric disorder. Control participants were matched with patients with bvFTD according to age [*t* (26) = .15, *P* = .88], sex [*X*^2^ (1, N = 28) = .08, *P* = .78] and educational level [*t* (26) = .14, *P* = .89]. The study was registered in Clinical Trials under the number NCT05642351 and was conducted in according to the Declaration of Helsinki. All patient and control participants provided informed consent. Ethical approval was obtained from the national French ethical committee (approval number: 2022-A02482-41).

### 2.2. Procedures

We evaluated general cognitive function with the Mini Mental State Exam.^[22]^ As expected, the patients scores were significantly lower (*M* = 19.95/30 points, *SD* = 3.36) than those of the control participants (*M* = 28.89, *SD* = .52) [*t* (26) = 10.19, *P* < .001]. We also assessed episodic memory^[23]^ by inviting participants to retain 16 words, each word depicting an item (e.g., geography) that belongs to a different semantic category (e.g., science). Afterwards, participants were allocated 2 minutes for free recall and the maximum number of retrieved words/16 was retained as their score on this test. The patients scores were significantly lower (*M* = 6.16 words, *SD* = 2.90) than those of the control participants (*M* = 11.24, *SD* = 2.97) [*t* (26) = 4.56, *P* < .001]. We finally assessed verbal fluency by inviting participants to generate words beginning with the letter P in 1 minute. The patients scores were significantly lower (*M* = 9.53 words, *SD* = 3.43) than those of the control participants (*M* = 17.73, *SD* = 4.32) [*t* (26) = 5.50, *P* < .001].

We implemented the “Who am I?” task, which was developed by Charlesworth, Allen, Havelka and Moulin^[5]^ for use in the general population and was later implemented in patients with Alzheimer’s Disease.^[24–26]^ In our study, participants were invited to produce as many verbal statements to the question “Who am I?” as possible. We explained that the task involved production of very short statements beginning with the phrase “I am.” We also explained that these statements should describe what the participants felt were essential in defining who they are. We further explained that statements should reflect enduring aspects of the identity. These aspects might include roles and traits (i.e., psychological or physical). A time limit of 1 minute was used and the duration was made explicit from the outset so participants could plan accordingly.

We coded data regarding the 3 categories: physical, social, and psychological self. In line with the procedures of Rathbone, Conway and Moulin,^[27]^ we defined physical statements as those describing attributes related to appearance (e.g., old, tall, fat…). We defined social statements as those describing social attributes or roles (e.g., grandmother, retired, volunteering). We defined psychological statements as those describing personality traits (e.g., honest, kind, friendly), or emotional states (e.g., anxious, happy, sad). In line with the recommendations of Rhee, Uleman, Lee, and Roman,^[28]^ were analyzed statements describing 2 self- components in terms of the basic unit. For instance, we attributed the statement “I am a gentle father” to social self as the information (“a father”) is the basic unit of information, rather than the evaluation (“gentle”). When responses depicted different closely related meanings (e.g., “I am cordial and kind”), only the first meaning was coded. We excluded repeated statements. We also excluded those not opening with “I am” (e.g., some participants said “I like to be gentle,” which was not taken into account). Statements beginning with “I was” or “I will” were also excluded as these sentences were seldom produced by patients with bvFTD. Data were processed by 2 independent raters and a post-coding comparison revealed 92% agreement between raters; discords were discussed until a consensus was reached.

### 2.3. Statistical analysis

The score in the “Who am I” task was the total number of statements, as well as the number of those that were attributed to physical, social, and psychological self. We compared differences on the production of “Who am I?” statements, as related to the 3 self-components (i.e., physical, social, and psychological self), between patients with bvFTD and control participants. We also compared the production of statements across the 3 self-components within each population. Because data was skewed [e.g., distribution of the physical statements in patients with bvFTD, *D* (13) = .75, *P* = .002] we used 2-tailed nonparametric tests. We provided effect size^[29]^ for nonparametric tests following the recommendations by Rosenthal and DiMatteo,^[30]^ and Ellis.^[31]^ We also carried out Spearman correlations to evaluate the relationship between “Who am I?” statements and cognitive tests separately for patients with bvTFD and control participants.

## 3. Results

### 3.1. Fewer “Who am I?” statements in patients with bvTFD than in controls

**Figure 1.**
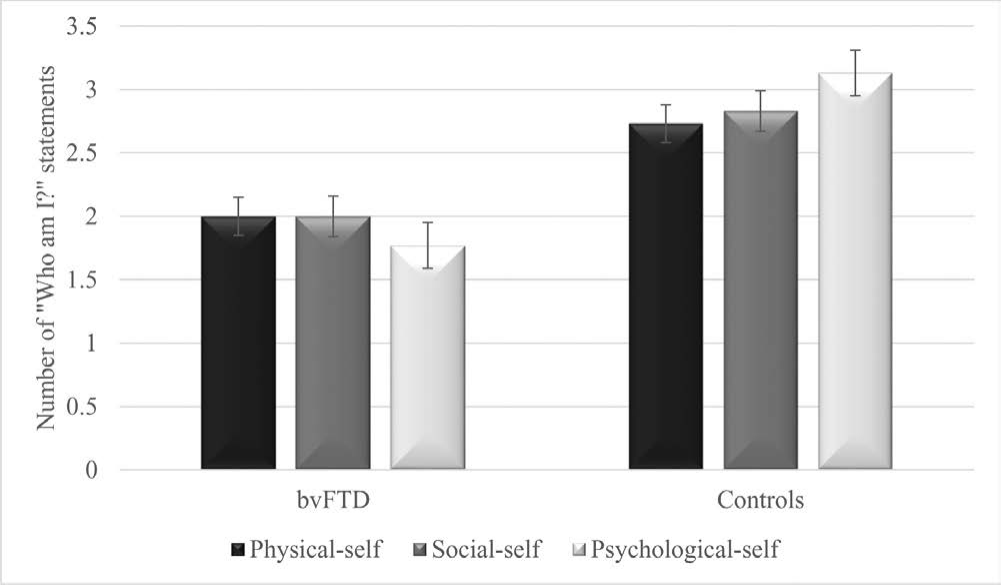
Number of “Who am I?” statements with regard to the 3 self- components in patients with behavioral variant frontotemporal dementia (bvFTD) and control participants. Error bars represent intervals of 95 % within-subjects confidence.

The production of “Who am I?” statements by group is illustrated in Figure 1. Mann-Whitney U tests revealed fewer total “Who am I?” statements in patients with bvFTD (*M* = 5.77, *SD* = 1.30) than in controls (*M* = 8.73, *SD* = 1.79) (Z = −3.74, *P* < .001, Cohen *d* = 2.00). The paucity of patients with bvFTD in generating “Who am I?” statements extended to all 3 categories: patients produced fewer statements describing physical self (*M* = 2.00, *SD* = .58) than controls (*M* = 2.73, *SD* = .88) (Z = −2.35, *P* = .019, Cohen *d* = .99), fewer statements describing social self (*M* = 2.00, *SD* = .71) than controls (*M* = 2.87, *SD =* .74) (Z = −2.68, *P* = .007, Cohen *d* = 1.17), as well as fewer statements describing psychological self (*M* = 1.77, *SD =* .72) than controls (*M* = 3.13, *SD =* .83) (Z = −3.58, *P* < .001, Cohen *d* = 1.85).

### 3.2. Similar production of statements about physical, social, and psychological self

Friedman tests revealed no significant differences between production of statements describing physical, social, and psychological self, in either patients with bvFTD [*X*^2^ (2, N = 13) = .74, *P* = .69, Cohen *d* = .35], or in controls [*X*^2^ (2, N = 15) = 2.48, *P* = .29, Cohen *d* = .49].

### 3.3. Significant correlations between “Who am I?” statements and verbal fluency

**Figure 2.**
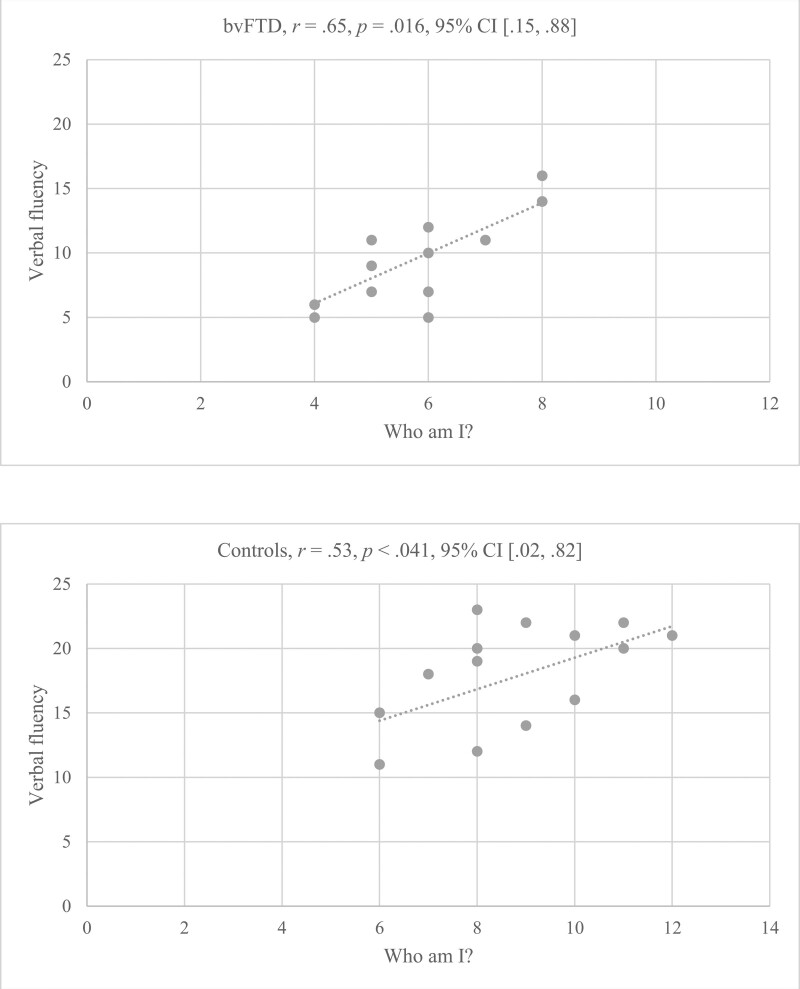
Correlations between total production of “Who am I?” statements and verbal fluency in patients with behavioral variant frontotemporal dementia (bvFTD) and control participants.

As shown in Figure 2, significant correlations were observed between the total number of “Who am I” statements and verbal fluency in both patients with bvFTD and controls. However, no significant correlations were observed between statements related to any of the 3 self-components (i.e., physical, social, and psychological self) and verbal fluency either in patients with bvTFD (*P* > .1) or in control participants (*P* > .1). In other words, verbal fluency was significantly correlated with the general production of “Who am I statements?,” but not with the production of statements related to any specific self-component. Moreover, no significant correlations were observed between either the total number or any specific categories of “Who am I” statements and scores for general cognitive ability or verbal episodic memory in any population (*P* > .1).

## 4. Discussion

In this paper, we assessed the sense of self in patients with bvTFD using the “Who am I?” task, which is based on the identical question. More specifically, we assessed how patients with bvFTD describe their physical, social, and psychological self. Analyses showed fewer statements related to all 3 self-categories in patients with bvFTD than in control participants. Another outcoming was the similar production of statements describing physical, social, and psychological self in patients with bvFTD and control participants. Finally, the total production of “Who am I?” statements was significantly positively correlated with verbal fluency, in both patients with bvTFD and control participants, but no significant correlations were observed with general cognitive ability or verbal episodic memory.

The first finding of our study was the fewer statements related to all 3 self-categories in patients with bvFTD than in control participants Our study thus demonstrates a diminished ability in patients with bvFTD to produce statements describing their self, and more specifically, a diminished ability to describe their physical, social, and psychological self. The diminished ability to produce “Who am I?” statements in bvFTD may mirror the diminished ability of patients with bvFTD to process self-images such as strengths, weaknesses, and possibilities for change, or even to put into action some positive self-images to cope with their illness. This finding is of clinical relevance because activating coping strategies and compensatory mechanisms typically require dealing with disease-related images (e.g., “I am patient with bvFTD”…). Thus, the diminished ability to produce “Who am I?” statements in patients with bvTFD may be associated with a difficulty to identify the impact of the disease on their sense of self, a difficulty that may result in diminished desire to deal with their disease-related identity and even the establishment of new self-images.

The diminished ability to produce “Who am I?” statements in patients with bvTFD may also reflect a hampered reflexive-self. A distinction has been made between the minimal-self and the reflexive-self, with the minimal-self referring to biological urges that make organisms reacting to their goals towards the world, and the reflexive-self referring to the consciousness of oneself as a subject of experience (i.e., the “I am” experience).^[32–35]^ Thus, the diminished ability to produce “Who am I?” statements in patients with bvTFD may reflect a diminished ability of patients to construct the consciousness experience of who they are now and even who they have been for most of their lives. Further, the diminished ability to produce “Who am I?” statements in patients with bvTFD may mirror a weakened identity in bvFTD, as the “Who am I?” task mirrors the ability to produce self-images that contains both factual and evaluative knowledge of identity.^[5]^ The disturbed identity in bvFTD has been assessed by Harris, Baird and Harris^[36]^ who invited family members of patients to describe the patients before and after the onset of the disease, with results demonstrating a disruption of the patients identity after the onset of the disease. Our study expands the study of Harris, Baird and Harris^[36]^ by relying on patients themselves to produce statements describing their identity.

Regarding the production of fewer statements describing physical self in patients with bvTFD compared to control participants, the disease may disturb the way patients view the nature or limits of their physical being. The disruption of the physical self in bvFTD may also reflect a hierarchical disruption in the sensory-self interaction, for instance, patients may suffer deficits in sensations, body recognition, and consequently, in self-body identification. Some support to this assumption can be found in the work of Feinberg^[37]^ who suggested that neurological disorders disturb the way patients perceive their own body. Regarding the production of fewer statements describing social self in patients, we suggest that the disease may affect the way patients perceive their social roles or even their interactions with others and vice-versa. This disrupted social perception of the self may support/be associated with the apathy in bvFTD. In other words, the hampered ability to construct images related to social self may underly/be associated with the typical pervasive decline in social motivated behaviors in bvFTD. Regarding the production of fewer statements describing psychological self in patients with bvTFD compared to control participants, we suggest that bvTFD may affect the way patients perceive and evaluate knowledge about their own personality traits.

Regarding the second main finding, that is, lack of significant differences between the number of statements describing physical, social, and psychological self-produced by patients with bvFTD, we suggest that the patients may show no preference, or even avoidance, of a particular self-component, probably because the disease results effects to a similar degree the processing of all 3 self-components. Regarding control participants, since they were independent and free of psychiatric and neurological disorders, they were likely to experience a balanced set of physical, social, and psychological traits and roles, resulting in the similar production of statements describing physical, social, and psychological self.

Regarding the third main findings, that is, the correlation between the general production of “Who am I?” statements and performance on the verbal fluency task. This correlation can be attributed to similarities between the “Who am I” and verbal fluency tasks, as both involve the production of verbal material to command and in a limited time interval. This significant correlation may also, somehow, be interpreted as mirroring the assumption that the lower “Who am I?” statements in patients with bvFTD compared to control participants may be attributed to the decreased verbal ability in the patients.

### 4.1. Limitations and recommendations

Regarding the lack of significant correlations between verbal fluency and each of the 3 self-categories considered independently, this can be attributed to the small number of statements within each self-category, which may have created a floor effect. Future research can address this limitation by allowing more time for production of the statements. Another suggestion for future research may be the assessment of future self-images (e.g., by inviting participants to provide statements beginning with “I will be”), as bvTFD has been associated with diminished future thinking.^[38]^ One further suggestion would be to assess pupil size on “Who am I?” task in light of research demonstrating how cognitive processing can be indexed by pupil size in patients with bvFTD^[39]^ as well as research demonstrating how verbal processing can be indexed by pupil size in general population.^[40,41]^ Finally, although one may argue that the sample size is small, we would like to emphasize that the Cohen *d* outcomes were fair, and even large for the Mann-Whitney *U* tests. Also, bvFTD is a rare neurological condition^[42]^ and recruitment of a large cohort of patients is not easily obtainable.

## 5. Conclusion

While research has extensively assessed the sense of self in bvFTD regarding anosognosia and self-consciousness, little attempt has been made to understand how the patients answer the basic, but fundamental, “Who am I?” quasi-metaphysical question. Using the “Who am I?” task, our study offers the first evidence of the diminished ability of patients with bvFTD to capture and describe factual and evaluative knowledge of themselves. By doing this, our study further sheds light on the hampered reflective consciousness of the self in patients with bvFTD. At the clinical level, our study paves the way towards the use of “Who am I?” as a simple and ecological tool allowing for the quantitative and qualitative exploration of physical, social, and psychological self in patients with bvFTD.

## Acknowledgments

MEH was supported by DISTALZ LABEX. DK was supported entirely by the Intramural Research Program of the National Institute on Aging, NIH.

## Author contributions

**Data curation:** Mohamad El Haj.

**Funding acquisition:** Dimitrios Kapogiannis.

**Investigation:** Mohamad El Haj, Claire Boutoleau Bretonnière.

**Methodology:** Mohamad El Haj.

**Resources:** Dimitrios Kapogiannis.

**Supervision:** Dimitrios Kapogiannis, Claire Boutoleau Bretonnière.

**Validation:** Claire Boutoleau Bretonnière.

**Writing – original draft:** Mohamad El Haj.

**Writing – review & editing:** Dimitrios Kapogiannis, Claire Boutoleau Bretonnière.
